# Different amounts of protein-bound citrulline and homocitrulline in foot joint tissues of a patient with anti-citrullinated protein antibody positive erosive rheumatoid arthritis

**DOI:** 10.1186/1479-5876-11-224

**Published:** 2013-09-23

**Authors:** Sanna Turunen, Marja-Kaisa Koivula, Jukka Melkko, Eeva Alasaarela, Petri Lehenkari, Juha Risteli

**Affiliations:** 1 Department of Clinical Chemistry, Institute of Diagnostics, University of Oulu, Oulu, Finland; 2 Northern Finland Laboratory Centre NordLab, Oulu University Hospital, Oulu, Finland; 3 Department of Pathology, Institute of Diagnostics, University of Oulu, Oulu, Finland; 4 Department of Pathology, Oulu University Hospital, Oulu, Finland; 5 Department of Internal Medicine, Division of Rheumatology, Oulu University Hospital, Oulu, Finland; 6 Department of Anatomy and Cell Biology, Institute of Biomedicine, University of Oulu, Oulu, Finland; 7 Surgery Clinic, Oulu University Hospital, Oulu, Finland

**Keywords:** Citrulline, Homocitrulline, Rheumatoid arthritis, Autoantibodies, Tissue, Carbamylation

## Abstract

**Background:**

Antibodies binding to citrullinated proteins are a frequent finding in rheumatoid arthritis patients and may precede the onset of clinical symptoms several years. The antibodies are a predisposing factor for bone erosions but their origin is unknown. In this study we analyze in detail the levels of protein bound citrulline and homocitrulline in several tissue samples of a single erosive arthritic surgery patient.

**Methods:**

Serum antibodies binding to CCP, MCV and citrulline- or homocitrulline-containing type I and II collagen carboxytelopeptides were measured. Tissue samples of a single RA patient, taken in two separate operations performed with two-year time span were hydrolyzed and analyzed for citrulline and homocitrulline content by HPLC.

**Results:**

Protein-bound citrulline and homocitrulline were found in several joint tissues of a RA patient with ACPA-positive erosive disease. The amount of homocitrulline stayed relatively constant between the different tissues. The amount of citrulline in erosive tissue was 3-times higher than in non-erosive tissue in the first operation. In the samples of the second operation 3-4-times higher mean amounts of citrulline were found in two out of the six tissues investigated.

**Conclusions:**

Homocitrulline is present in rheumatoid nodule together with citrulline. There is more variation in the amount of citrulline than in the amount of homocitrulline between the tissues. The tissue sample containing the most citrulline was the most erosive.

## Background

Antibodies binding to citrullinated proteins are a frequent finding in rheumatoid arthritis (RA) patients and may precede the onset of clinical symptoms several years [1]. The anti-citrullinated protein antibodies (ACPA) are a predisposing factor for bone erosions in RA patients [2]. The ACPA antibody repertoire of RA patients has been quite specifically characterized [3-5] and possible candidate antigens for these antibodies have also been demonstrated in several studies [6-8]. Antibodies binding to carbamylated proteins have been found recently in RA sera [9] but the presence of homocitrulline-containing proteins in RA tissues have not been demonstrated yet. Recent studies on RA have suggested that the original antigen of ACPA could be in the lungs [10] or in the gingiva [11]. The inflammation of the joints is still the most prominent symptom and chronic joint inflammation resistant to medication is treated surgically. In this study we analyzed for the first time in detail the levels of protein bound citrulline and homocitrulline in several tissue samples of a single erosive arthritic surgery patient.

## Patient and methods

### Patient

**Figure 1:**
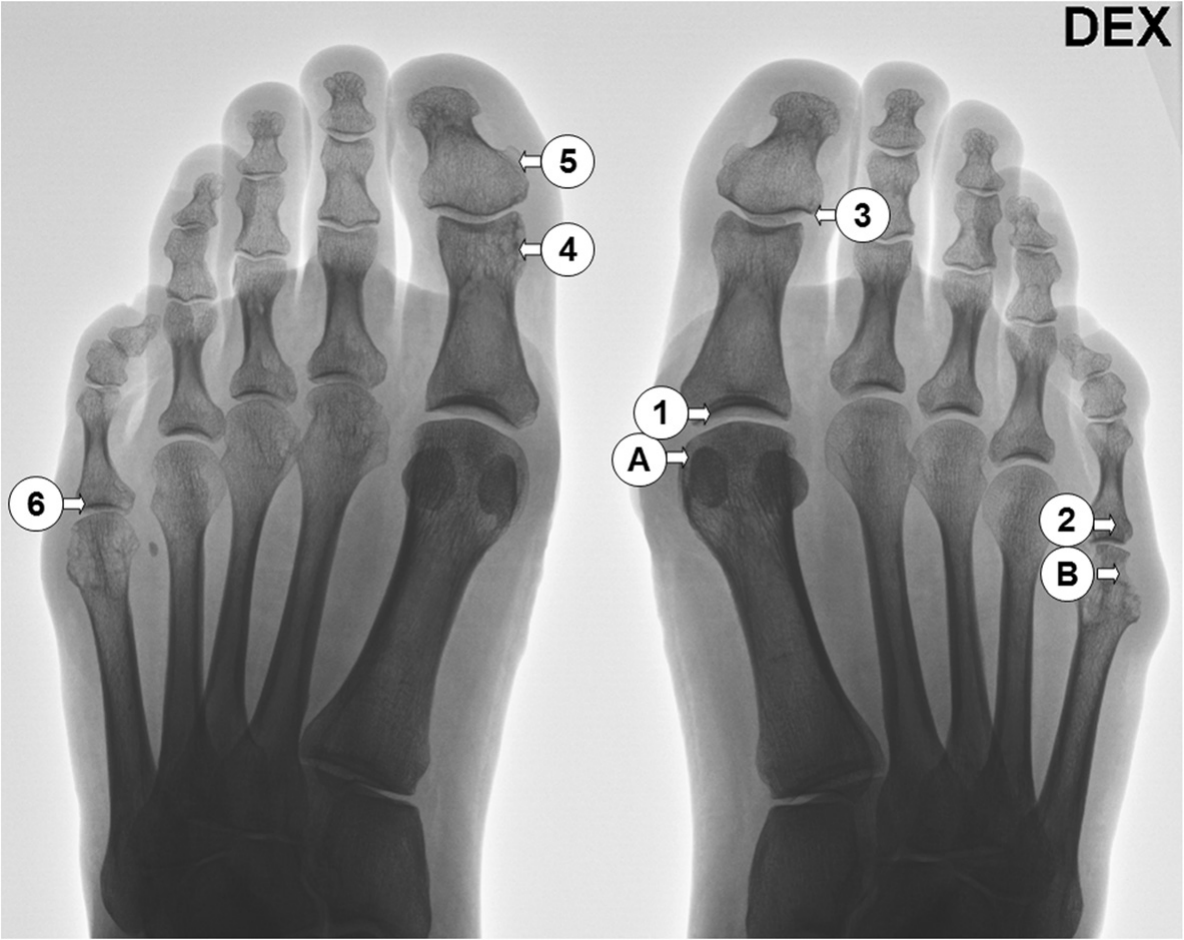
**The patient radiograph taken before the first surgery.** The locations of the samples taken are presented by **A** and **B** for the first operation and numbered from 1–6 for the second operation.

47-year-old female RA patient with a disease history of 10 years, at the time of the first operation, described here due to difficult and resistant local symptoms at right MTP1 and MTP5 joints. Prior to this several operations were performed to remove synovitis and rheumatoid nodules from both hands. The patient used multiple medication, that included natriumaurothiomalate 50 mg monthly, methotrexate 12.5 mg once weekly, leflunomide 20 mg daily and prednisolone 2.5 mg daily. Tissue samples were collected from two separate operations with two-year time span (Figure 1A and B in the first operation and 1–6 in the second operation).

### Histology

Samples were cut from the tissues (A, B and 1–6) frozen at −70°C, fixed in formalin and embedded in paraffin. 5 μm sections were mounted on glass slides and stained with hematoxyline-eosine.

### Assessment of autoantibodies in the patient serum

Serum sample obtained at the time of the second surgery was analyzed for binding anti-CCP (Immunoscan RA (Mark 2) Euro Diagnostica, Malmö, Sweden) and anti-MCV (ORG 546, ORGANTEC Diagnostica GmbH, Mainz, Germany). Also antibodies binding to citrulline or homocitrulline containing collagen type I and II carboxytelopeptides were measured from the serum as described previously [12,13].

### Detection of protein-bound citrulline and homocitrulline on high-performance liquid chromatography (HPLC)

Six approximately 10 mg (wet weight) samples were cut from each tissue. The samples were dialyzed 16 h against 0.2 M NH_4_HCO_3_, pH 7.4 to separate free amino acids from protein-bound ones and freeze-dried, then rehydrated in distilled H_2_O, freeze-dried and hydrolyzed in 6 M HCl at 110°C for 16 h and freeze-dried. The samples were chemically modified and analyzed on HPLC as reported previously [12]. The values for citrulline and homocitrulline in the sample were read from the automatically integrated peak area. The sample mass variation was taken in to account by using a correction coefficient.

### Statistical analysis

The peak areas of citrulline and homocitrulline between tissues were assessed by independent samples Mann–Whitney U test for the first operation tissues (n=2) and by Kruskal-Wallis test for the second operation tissues (n=6) using Statistical Package for Social Science (SPSS) for Windows version 20 (IBM SPSS Statistics, Armonk, New York, USA). The differences were considered significant at p < 0.05.

### Ethical considerations

The research plan was approved by the Ethics Committee of Oulu University Hospital, the guidelines of the Declaration of Helsinki were followed and an informed consent was signed by the patient.

## Results

**Figure 2:**
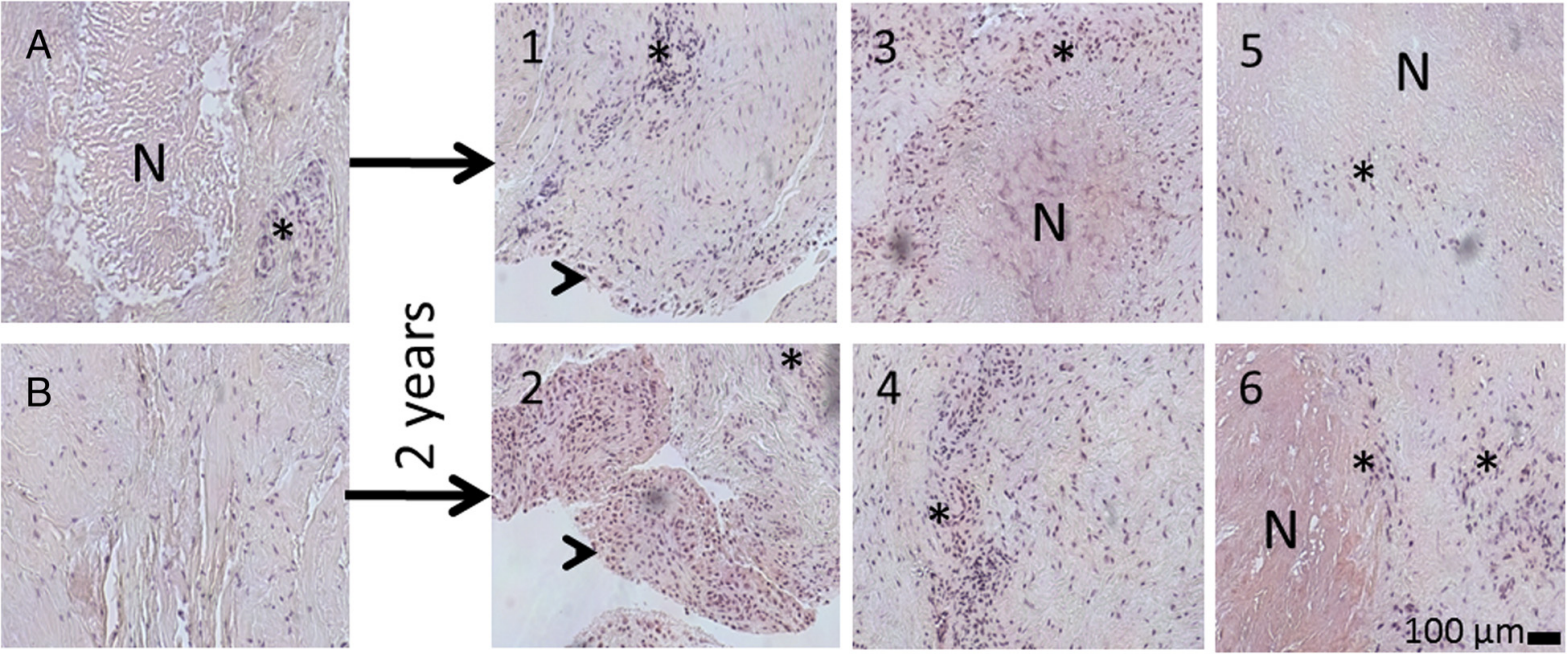
**Histological images of removed tissues.** Numbers correspond those shown in Figure 1. Interval between operations was 2 years, **A** and 1, **B** and 2 are samples from same anatomical site, respectively. Arrowheads indicate epithelial tissue, * indicates inflammatory cells, mostly macrophages, N indicates necrosis in the rheumatoid nodulus.

The patient was positive for antibodies binding to CCP (800.5 U/ml) and MCV (55.9 U/ml) but no antibodies binding to citrulline- or homocitrulline-containing type I and II collagen carboxytelopeptides were detected. Tissue samples A and B (Figure 1) were collected during the first operative treatment to remove rheumatoid synovitis. Tissue B was from an area where the tissue was both visually and radiologically eroded. In histological studies the tissues were classified as rheumatoid nodules characterized by necrotic tissue surrounded by palisading macrophages. RA tissue consists of a mixture of various tissues, such as vascular, epithelial, different types of fibrotic and necrotic tissues. All samples were adjacent to synovium, hence some synovial fragments were present, as shown in Figure 2.

**Figure 3:**
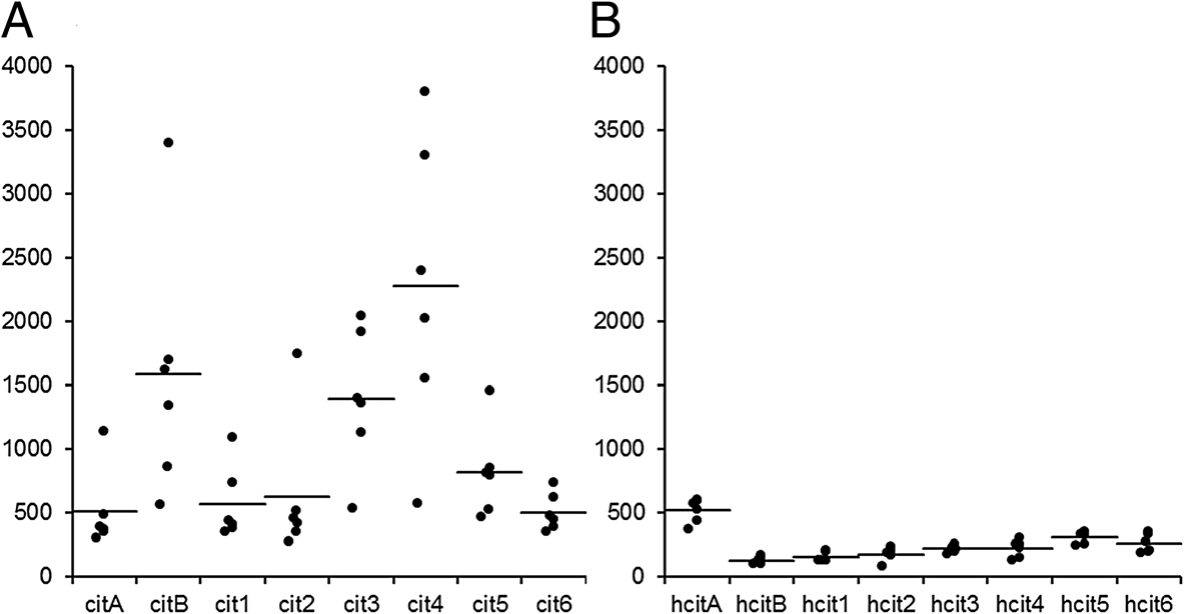
**Results of the HPLC-analysis of citrulline and homocitrulline.** The auto-integrated HPLC peak areas of **A)** citrulline and **B)** homocitrulline in the six samples analyzed from each of the eight removed foot joint tissues. In the first operation tissue **A** was non-erosive and tissue **B** was from an erosive location.

In the HPLC analysis of the first operation tissue samples the mean level of homocitrulline was lower and mean level of citrulline 3-times higher in the erosive B compared to the non-erosive tissue A (Figure 3). In the Mann–Whitney independent samples U test both the differences in citrulline and in homocitrulline between tissues were statistically significant (p <0.001).

The second operation was scheduled after 2-years follow-up due to synovitis relapse. A more radical operation was programmed in consensus with the patient due to extensive synovial tissue growth in multiple sites and the aim was to remove all active synovitis and hence 6 separate cuts were made. Representative samples were taken from metatarsal joints (Figure 1: 1, 2, 6) and from interphalangeal joints (Figure 1: 3, 4, 5). In HPLC analysis homocitrulline and citrulline were present in all samples. The level of homocitrulline stayed more constant between both operations and the different samples compared to citrulline (Figure 3). In the Kruskal-Wallis independent sample tests both the differences in citrulline and in homocitrulline levels between tissues of the second operation were statistically significant p = 0.001 and p = 0.003 respectively. In the pairwise comparison tissue number 5 differed statistically from tissues 1 and 2 with greater amount of homocitrulline (p = 0.003 and 0.022). The amount of citrulline in tissue number 4 was statistically greater than in tissues 1, 2 and 6 (p = 0.020, 0.024 and 0.029, respectively). Clinically the patient recovered from both surgeries without complications and the patient reported a significant reduction in RA symptoms and arthrodesis of the right MTP 1 joint was cancelled.

## Discussion

In this study, we report the detailed analysis of protein-bound citrulline and homocitrulline in the RA affected tissues of a severely RA symptomatic patient. Citrulline and in experimental model also homocitrulline injected with adjuvant [12] can induce antibodies against citrullinated proteins that are a predisposing factor for erosive RA. We hypothesized that our systemically ACPA positive patient could have citrullinated proteins locally at the symptomatic site of intended surgery. The erosions were evident already in the first radiograph and repeated sampling from surgically removed synovitis tissue revealed citrullinated proteins in the tissues. Homocitrulline was present in all samples but the level of homocitrulline was low compared to citrulline and stayed more constant between both operations and different tissues (Figure 3A and B). In the postoperative surgical interview the subjective general and especially the feet symptoms caused by RA were alleviated by the removal of the diseased tissue. However, it is of interest that the clinical symptoms were only alleviated after the second, more thorough operation. This suggests that although the primary induction of the ACPA might be elsewhere the removal of the citrulline and homocitrulline containing tissues affects the local disease activity. The local presentation of the citrullinated antigens could be a reason for erosion. Lately osteoclastogenesis and bone loss induced by antibodies binding to citrullinated vimentin was demonstrated [14]. Interestingly the PAD expression pattern in osteoclasts is similar that is found in the macrophages of the inflamed RA synovium [15,16]. The bone loss in RA is not exclusive consequence of synovitis, but neither ACPA alone is enough to cause classical bone erosions [17].

Antibodies binding to carbamylated proteins were recently found in RA patient sera [9]. The presence of homocitrulline, the product of lysine carbamylation, however, has not been demonstrated earlier in RA tissues. The relevance of homocitrulline to RA pathogenesis is not clear, but at least in animal model protein-bound homocitrulline injected with adjuvant was able to induce antibodies binding to citrullinated proteins [12]. Carbamylation can be caused by inflammation [18] or ageing of tissues [19]. Therefore the differences between our samples could be explained by two alternative ways explaining either the low or the high homocitrulline level. High level of homocitrulline could be a consequence of the chronic inflammation related to RA. On the other hand a low level of homocitrulline could be explained by repair that creates the RA synovitis tissue as the newly formed tissue has not gained age-related posttranslational modifications like the lysine carbamylation to homocitrulline. The highest homocitrulline value was from MTP 1 joint (A) that was the most symptomatic and the primary cause for the first surgical intervention. Bearing this in mind, tissue B was highly interesting because it had the lowest level of homocitrulline and high level of citrulline and the tissue was highly erosive. This would suggest presence of an active disease process with increased citrullination in the newly formed tissue. Recently, neutrophil extracellular traps were suggested as the source of citrullinated antigens in RA [20]. We could not detect any neutrophils in the basic histological analysis of the nodules, only macrophages and a few plasma cells, though the presence of citrulline in the chemical analysis was evident.

As limitations for this case report, it should be noted that the results gained do not necessarily reflect the situation in other tissue specimens, such as larger joints or in other RA patients. Also it is notable, that the amount of citrulline and homocitrulline in each sample was affected by the exact location, where the 10 mg sample was taken because all specimens were analyzed separately. Rheumatoid nodules are not uniform, they consisted of various amounts of necrosis that was surrounded by different amount of mainly macrophages and other inflammatory cells, such as lymphocytes so even the same tissue contained areas that had different levels of each amino acid. For this same reason the appearance of the tissue (inflammation and necrosis) could not be correlated with the HPLC analysis results.

## Conclusions

This is the first study analyzing in detail the significant local differences in citrulline and homocitrulline content of several tissues of a single ACPA-positive patient with erosive RA and showing the presence of homocitrulline in rheumatoid nodule tissues.

## Abbreviations

RA: Rheumatoid arthritis; ACPA: Anti-citrullinated protein antibodies; Anti-CCP: Anti-cyclic citrullinated peptide (clinical assay); Anti-MCV: Anti-mutated citrullinated vimentin (clinical assay); HPLC: High performance liquid chromathography; MTP: Metatarsophalangeal (joint).

## Competing interests

The authors declare that they have no competing interests.

## Authors’ contributions

ST, MKK, PL and JR contributed to study design. PL operated the patient and collected samples. ST carried out the immunoassays and HPLC analysis. JM conducted the histological analysis. EA did the rheumatological evaluation of the patient. ST and PL drafted the manuscript. All authors revised the manuscript and read and approved the final version.

## References

[B1] Koivula MK Heliovaara M Ramberg J Knekt P Rissanen H Palosuo T Risteli J Autoantibodies binding to citrullinated telopeptide of type II collagen and to cyclic citrullinated peptides predict synergistically the development of seropositive rheumatoid arthritis Ann Rheum Dis 2007 66 1450 1455 10.1136/ard.2006.062919 17472989 PMC2111613

[B2] Lundberg K Nijenhuis S Vossenaar ER Palmblad K van Venrooij WJ Klareskog L Zendman AJ Harris HE Citrullinated proteins have increased immunogenicity and arthritogenicity and their presence in arthritic joints correlates with disease severity Arthritis Res Ther 2005 7 R458 R467 10.1186/ar1697 15899032 PMC1174941

[B3] Nielen MM van Schaardenburg D Reesink HW van de Stadt RJ van der Horst-Bruinsma IE de Koning MH Habibuw MR Vandenbroucke JP Dijkmans BA Specific autoantibodies precede the symptoms of rheumatoid arthritis: a study of serial measurements in blood donors Arthritis Rheum 2004 50 380 386 10.1002/art.20018 14872479

[B4] Suwannalai P Trouw LA Toes RE Huizinga TW Anti-citrullinated protein antibodies (ACPA) in early rheumatoid arthritis Mod Rheumatol 2012 22 15 20 10.1007/s10165-011-0486-8 21732051

[B5] van de Stadt LA de Koning MH van de Stadt RJ Wolbink G Dijkmans BA Hamann D van Schaardenburg D Development of the anti-citrullinated protein antibody repertoire prior to the onset of rheumatoid arthritis Arthritis Rheum 2011 63 3226 3233 10.1002/art.30537 21792832

[B6] Lundberg K Kinloch A Fisher BA Wegner N Wait R Charles P Mikuls TR Venables PJ Antibodies to citrullinated alpha-enolase peptide 1 are specific for rheumatoid arthritis and cross-react with bacterial enolase Arthritis Rheum 2008 58 3009 3019 10.1002/art.23936 18821669

[B7] Koivula MK Aman S Karjalainen A Hakala M Risteli J Are there autoantibodies reacting against citrullinated peptides derived from type I and type II collagens in patients with rheumatoid arthritis? Ann Rheum Dis 2005 64 1443 1450 10.1136/ard.2004.031211 16162901 PMC1755220

[B8] Masson-Bessiere C Sebbag M Girbal-Neuhauser E Nogueira L Vincent C Senshu T Serre G The major synovial targets of the rheumatoid arthritis-specific antifilaggrin autoantibodies are deiminated forms of the alpha- and beta-chains of fibrin J Immunol 2001 166 4177 4184 11238669 10.4049/jimmunol.166.6.4177

[B9] Shi J Knevel R Suwannalai P van der Linden MP Janssen GM van Veelen PA Levarht NE van der Helm-van Mil AH Cerami A Huizinga TW Toes RE Trouw LA Autoantibodies recognizing carbamylated proteins are present in sera of patients with rheumatoid arthritis and predict joint damage Proc Natl Acad Sci U S A 2011 108 17372 17377 10.1073/pnas.1114465108 21987802 PMC3198314

[B10] Klareskog L Stolt P Lundberg K Kallberg H Bengtsson C Grunewald J Ronnelid J Harris HE Ulfgren AK Rantapaa-Dahlqvist S Eklund A Padyukov L Alfredsson L A new model for an etiology of rheumatoid arthritis: smoking may trigger HLA-DR (shared epitope)-restricted immune reactions to autoantigens modified by citrullination Arthritis Rheum 2006 54 38 46 10.1002/art.21575 16385494

[B11] Nesse W Westra J van der Wal JE Abbas F Nicholas AP Vissink A Brouwer E The periodontium of periodontitis patients contains citrullinated proteins which may play a role in ACPA (anti-citrullinated protein antibody) formation J Clin Periodontol 2012 39 599 607 10.1111/j.1600-051X.2012.01885.x 22530757

[B12] Turunen S Koivula MK Risteli L Risteli J Anticitrulline antibodies can be caused by homocitrulline-containing proteins in rabbits Arthritis Rheum 2010 62 3345 3352 10.1002/art.27644 20617522

[B13] Koivula MK Aman S Alasaarela E Karjalainen A Hakala M Risteli J Inhibitory characteristics of citrullinated telopeptides of type I and II collagens for autoantibody binding in patients with rheumatoid arthritis Rheumatology (Oxford) 2006 45 1364 1369 10.1093/rheumatology/kel113 16632481

[B14] Harre U Georgess D Bang H Bozec A Axmann R Ossipova E Jakobsson PJ Baum W Nimmerjahn F Szarka E Sarmay G Krumbholz G Neumann E Toes R Scherer HU Catrina AI Klareskog L Jurdic P Schett G Induction of osteoclastogenesis and bone loss by human autoantibodies against citrullinated vimentin J Clin Invest 2012 122 1791 1802 10.1172/JCI60975 22505457 PMC3336988

[B15] Foulquier C Sebbag M Clavel C Chapuy-Regaud S Al Badine R Mechin MC Vincent C Nachat R Yamada M Takahara H Simon M Guerrin M Serre G Peptidyl arginine deiminase type 2 (PAD-2) and PAD-4 but not PAD-1, PAD-3, and PAD-6 are expressed in rheumatoid arthritis synovium in close association with tissue inflammation Arthritis Rheum 2007 56 3541 3553 10.1002/art.22983 17968929

[B16] Chang X Yamada R Suzuki A Sawada T Yoshino S Tokuhiro S Yamamoto K Localization of peptidylarginine deiminase 4 (PADI4) and citrullinated protein in synovial tissue of rheumatoid arthritis Rheumatology (Oxford) 2005 44 40 50 10.1093/rheumatology/keh414 15466895

[B17] Kleyer A Finzel S Rech J Manger B Krieter M Faustini F Araujo E Hueber AJ Harre U Engelke K Schett G Bone loss before the clinical onset of rheumatoid arthritis in subjects with anticitrullinated protein antibodies Ann Rheum Dis 2013 Published online first, doi:10.1136/annrheumdis-2012-202958 10.1136/annrheumdis-2012-202958 23520034

[B18] Wang Z Nicholls SJ Rodriguez ER Kummu O Horkko S Barnard J Reynolds WF Topol EJ DiDonato JA Hazen SL Protein carbamylation links inflammation, smoking, uremia and atherogenesis Nat Med 2007 13 1176 1184 10.1038/nm1637 17828273

[B19] Jaisson S Gillery P Evaluation of nonenzymatic posttranslational modification-derived products as biomarkers of molecular aging of proteins Clin Chem 2010 56 1401 1412 10.1373/clinchem.2010.145201 20562349

[B20] Khandpur R Carmona-Rivera C Vivekanandan-Giri A Gizinski A Yalavarthi S Knight JS Friday S Li S Patel RM Subramanian V Thompson P Chen P Fox DA Pennathur S Kaplan MJ NETs Are a Source of Citrullinated Autoantigens and Stimulate Inflammatory Responses in Rheumatoid Arthritis Sci Transl Med 2013 5 178ra40 10.1126/scitranslmed.3005580 23536012 PMC3727661

